# Regulation of the Single Polar Flagellar Biogenesis

**DOI:** 10.3390/biom10040533

**Published:** 2020-04-01

**Authors:** Seiji Kojima, Hiroyuki Terashima, Michio Homma

**Affiliations:** Division of Biological Science, Graduate School of Science, Nagoya University, Chikusa-Ku, Nagoya 464-8602, Japan; terashima.hiroyuki@h.mbox.nagoya-u.ac.jp (H.T.); g44416a@cc.nagoya-u.ac.jp (M.H.)

**Keywords:** polar flagellum, FlhF, FlhG, HubP, FlaK, SflA, protein localization, ATPase, GTPase

## Abstract

Some bacterial species, such as the marine bacterium *Vibrio alginolyticus,* have a single polar flagellum that allows it to swim in liquid environments. Two regulators, FlhF and FlhG, function antagonistically to generate only one flagellum at the cell pole. FlhF, a signal recognition particle (SRP)-type guanosine triphosphate (GTP)ase, works as a positive regulator for flagellar biogenesis and determines the location of flagellar assembly at the pole, whereas FlhG, a MinD-type ATPase, works as a negative regulator that inhibits flagellar formation. FlhF intrinsically localizes at the cell pole, and guanosine triphosphate (GTP) binding to FlhF is critical for its polar localization and flagellation. FlhG also localizes at the cell pole via the polar landmark protein HubP to directly inhibit FlhF function at the cell pole, and this localization depends on ATP binding to FlhG. However, the detailed regulatory mechanisms involved, played by FlhF and FlhG as the major factors, remain largely unknown. This article reviews recent studies that highlight the post-translational regulation mechanism that allows the synthesis of only a single flagellum at the cell pole.

## 1. Introduction

**Figure 1:**
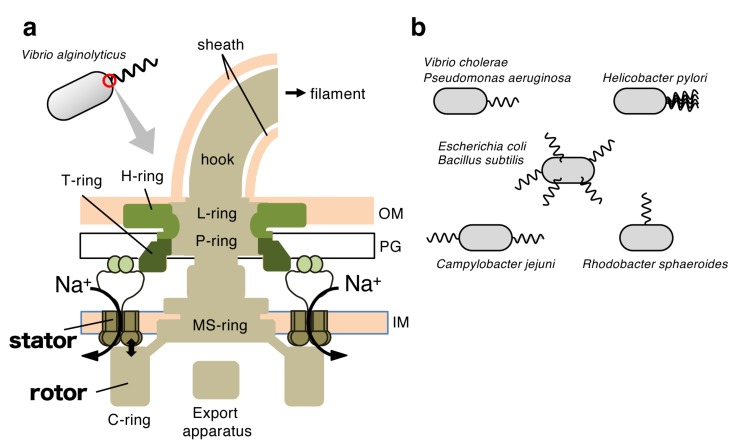
The flagellum is a bacterial motility organ. (**a**) Schematic of the polar flagellar motor of *Vibrio alginolyticus*. The rotor-stator interaction that couples with sodium ion influx through the stator channel generates motor torque. OM, outer membrane; IM, inner membrane; PG, peptidoglycan layer. (**b**) The number and location of flagella vary among bacterial species.

Motility is a fundamental function of bacteria required for survival in response to environmental changes. When bacteria encounter deleterious conditions, they must move out from such environments and seek more favorable ones. To achieve this cell movement (swimming in a liquid [1] or swarming on a surface [2]), many motile bacteria use the flagellum, their motility machinery [1,3]. The bacterial flagellum is structurally, functionally, and evolutionally distinct from its eukaryotic counterpart. The flagellum is extended from the cell body and consists of three parts: the filament (helical propeller), the hook (universal joint), and the basal body (rotary motor) (Figure 1a). Reversible rotation of the long helical filament thrusts the cell body forward or backward, and is driven by the membrane-embedded rotary motor at its base [4,5,6]. The energy source of the flagellar motor is the electrochemical gradient of specific ions (in most cases H^+^ or Na^+^) across the inner membrane. The motor is composed of a rotary part (the rotor) and energy-converting multiple stator units that surround the rotor. The motor torque is generated by rotor–stator interactions that couple to the ion influx through the stator channel [7,8]. Genetic, biochemical, and biophysical studies have unveiled the many protein components involved in flagellar function, their locations in the motor, and the rotational properties of the motor. However, the molecular mechanism of energy conversion during flagellar rotation has not yet been elucidated. For reviews of the rotary mechanism of the flagellar motor, please see references recently published elsewhere [9,10]. 

To achieve the best motility performance in a wide variety of habitats, bacteria have developed their own flagellar systems located at specific position(s) on the bacterial cells with suitable numbers, and the numbers and positions of flagella vary widely among bacterial species (Figure 1b) [11]. *Escherichia coli*, *Bacillus subtilis*, and *Salmonella enterica* have multiple peritrichous flagella and they form flagellar bundles to swim forward [9]. *Campylobacter jejuni* has bipolar flagella (one at each pole) [12], *Helicobacter pylori* has multiple polar flagella at one pole [13], and *Rhodobacter sphaeroides* has a single medially located flagellum [14]. *Pseudomonas aeruginosa* [15] and *Vibrio cholerae* [16] have a single polar flagellum at the cell pole. Surprisingly, the spirochete *Borrelia burgdorferi* has 7-11 periplasmic flagella [17,18] and *Leptospira biflexa* [19] possess two periplasmic flagella near each end of the cell body. In some species, two distinct types of flagella are produced. Well-known examples are *Vibrio alginolyticus* and *Vibrio parahaemolyticus*, which have a single sheathed polar flagellum suitable for swimming motility when grown in a liquid environment, but when they are grown on a surface, numerous lateral (peritrichous) flagella suitable for swarming motility are induced in response to the increased viscosity of the surrounding environment [20,21]. *Shewanella putrefaciens* CN-32 also possesses a two flagellar system, and the primary system generates a single polar flagellum, whereas a secondary flagellum is formed at a lateral position in subpopulations cultivated in complex medium [22]. In all cases, motility is impaired by mutations that cause defects in spatial and/or numerical control of flagella, indicating that the number and placement of flagella are precisely regulated to optimize motility under the environmental conditions of each bacterium [11].

It has been known that some species of bacteria have a single flagellum at their cell pole for motility. Then, one simple question arises—how do they generate only a single flagellum at the cell pole? In this regard, the marine bacterium *Vibrio alginolyticus*, which generates a single flagellum at its cell pole in a liquid environment [23], is a good model organism because genetic, biochemical, and structural analyses of its polar flagellar system have been extensively studied [4]. Moreover, the filament of the polar flagellum is covered with a membranous sheath contiguous with the outer membrane of the bacterial cell [24], and because of its thickness it can be easily observed using high intensity dark-field microscopy [25]. Using this bacterium, our group has reported that two key factors, FlhF and FlhG, play major roles in the regulation of polar flagellar number and placement. Here, we summarize our recent studies that have characterized the mechanisms by which FlhF and FlhG work to generate only a single polar flagellum in *Vibrio alginolyticus*, together with historical and recent insights obtained from other species. We also introduce other regulatory factors, HubP and SflA, which are involved in the biogenesis of polar flagella. It should be noted that the lateral flagella of *Vibrio alginolyticus*, which are induced in a viscous environment [20,26], are not the focus of this review. The unsheathed, thinner filament of lateral flagella is composed of components distinct from those of the polar flagella. All of our studies described in this review used bacterial strains that do not produce lateral flagella (VIO5 [27] and its derivatives). 

## 2. FlhF and FlhG Regulate Flagellar Number and Placement

FlhF and FlhG have been reported as factors that regulate the number and position of polar flagella in *Pseudomonas aeruginsa* and *Pseudomonas putida* [28,29], *Campylobacter jejuni* [12], *Shewanella putrefaciens* CN-32 [30], *Shewanella oneidensis* [31], *Vibrio cholerae* [16], and *Vibrio alginolyticus* [32]. In the year 2000, FlhF was first identified as a factor involved in the starvation survival of *Pseudomonas putida* [29]. Sequence analysis of the transposon mutant (MK107) that was impaired in stationary phase survival appeared to have a transposon Tn*5* insertion in *flhF*. Because *flhF* is flanked by known flagellar genes, it was implicated in flagellar biogenesis. Indeed, MK107 did not spread in soft agar plates although its active motility was observed using phase-contrast microscopy. Electron microscopy showed that unlike the wild-type strain, the flagella of MK107 were randomly distributed on the cell surface. Overproduction of FlhF from the plasmid caused the production of a large number of polar flagella. Therefore, FlhF promotes flagellar biosynthesis at the cell pole, and positively regulates the number of flagella [29]. Meanwhile, genome sequence analysis of *Pseudomonas aeruginosa* revealed that *fleN* (*flhG*) is also implicated in flagellar biogenesis. *fleN* is flanked by *flhF* and *fliA*, and genetic inactivation of *fleN* by inserting a gentamicin cassette resulted in the productions of multiple flagella at the cell pole. Therefore, FleN negatively regulates the number of polar flagella [33]. 

**Figure 2:**
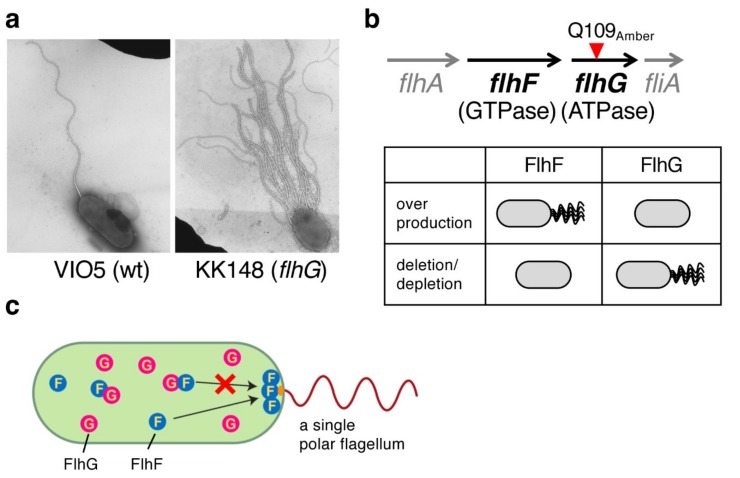
FlhF and FlhG regulate the number of polar flagella in *Vibrio alginolyticus*. (**a**) Electron micrographs of *V. alginolyticus* strain VIO5 (wild-type for polar flagellation) and KK148 (*flhG* mutant of VIO5). (**b**) The mutation site of KK148 (upper panel) and flagellar organisation of FlhF and FlhG variants of *Vibrio alginolyticus* (lower panel). The mutation was mapped on *flhG* (Q109Amber), which forms an operon with *flhF*. FlhF and FlhG work antagonistically. Their overproduction or deletion/depletion confers opposite phenotypes in *Vibrio alginolyticus*. (**c**) Model for the regulation of polar flagella by FlhF and FlhG proposed in [34]. In this model, FlhF localizes at the cell pole and determines the location of flagellation. FlhG interacts with FlhF to prevent its polar localization, and thereby negatively regulates the number of flagella.

In 2006, we reported that FlhF and FlhG also regulate the number of polar flagella in *Vibrio alginolyticus* [32]. During the screening of mutants defective in polar flagellar motility in soft agar plates, a mutant (KK148) was accidently isolated that had a large number of flagella at one pole (Figure 2a). KK148 formed a reduced motility ring compared to the wild-type strain VIO5, and an abnormal swimming behavior, caused by entangled multiple flagella, was observed for most cells using dark-field microscopy. Analysis of the *V. alginolyticus* genome revealed that *flhF* is flanked by *flhA* and *flhG* in the polar flagellar gene locus, and the mutation of KK148 was mapped to *flhG* (Gln109Amber, Figure 2b). Deletion studies revealed that the loss of FlhF resulted in a nonflagellated phenotype, whereas the loss of FlhG caused hyperflagellation (Figure 2b). Conversely, the overproduction of FlhF generated multiple polar flagella, but the overproduction of FlhG inhibited polar flagellation (Figure 2b). Therefore, similar to *Pseudomonas* spp., FlhF and FlhG function as positive and negative regulators of the number of flagella in *V. alginolyticus* [34], respectively. Because the deletion of both *flhF* and *flhG* confers a non-flagellated phenotype for most cells and FlhG mutants still form flagella at their cell pole, FlhF determines the flagellar placement at the cell pole [34]. 

Phenotypic analysis revealed that FlhF and FlhG work antagonistically to generate a single polar flagellum at the cell pole, but how do they achieve that? To determine the process involved, green fluorescent protein (GFP) was fused to the C-terminus of FlhF or FlhG, and their subcellular localization was observed [34]. Neither FlhF nor FlhG have a transmembrane segment and are thus expected to be cytoplasmic proteins. The fluorescent signal of FlhF-GFP was observed throughout the cytoplasm and most cells showed a fluorescent dot at the flagellated cell pole. This polar localization was observed more strongly in the absence of FlhG, in which multiple polar flagella were generated. Meanwhile, FlhG-GFP also diffused in the cytoplasm and its polar localization, which appeared to be independent of FlhF, was observed in ≈30% of cells. Because FlhG was immunoprecipitated by an anti-FlhF antibody from the cytoplasmic fraction, the first model proposed was that FlhF localization at the cell pole determines the polar localization and production of a flagellum, FlhG interacts with FlhF to prevent FlhF from localizing at the cell pole, and thus FlhG negatively regulates the number of flagella in *V. alginolyticus* (Figure 2c) [34]. 

It should be noted that the peritrichously flagellated bacterium *Bacillus subtilis* also produces both FlhF and FlhG. The deletion of *flhF* does not affect motility, but the basal body is formed at random positions compared to the wild-type strain. The deletion of *flhG* does not affect motility but causes the aggregation of basal bodies in the cell. FlhF and FlhG in *B. subtilis* seem to be important for the optimized spatial positioning of flagella with a grid-like pattern [35].

## 3. FlhF Is a SRP-Type GTPase

**Figure 3:**
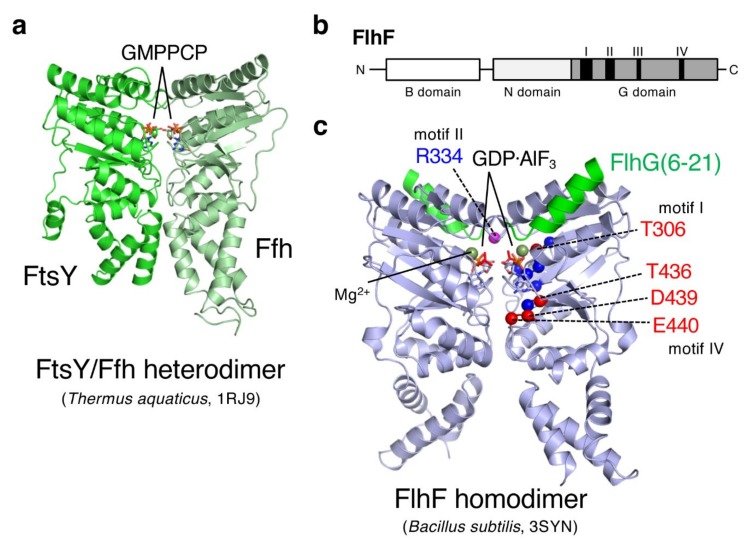
FlhF is a signal recognition particle (SRP)-type guanosine triphosphate (GTP)ase. (**a**) Crystal structure of the FtsY/Ffh heterodimer from *Thermus aquaticus* (PDB ID 1RJ9 [37]). The non-hydrolyzable guanosine triphosphate (GTP) analog β,γ-methyleneguanosine 5′-triphosphate (GMPPCP) stabilizes the heterodimer, and complex formation aligns the two molecules of this GTP analog in the composite active site. (**b**) Domain structure of FlhF proteins. *Bacillus subtilis* FlhF consists of 366 amino acids (41 kDa) with a smaller B domain than *Vibrio alginolyticus* FlhF (505 amino acids, 57 kDa). FlhF is composed of the function-unknown B domain, the regulatory N domain, and the G domain that contains the GTPase motif (I-IV). (**c**) Crystal structure of the NG domain homodimer from *Bacillus subtilis* FlhF in complex with the peptide containing N-terminal 23 residues of FlhG (PDB ID 3SYN [40]). FlhF is shown in light blue, and the FlhG peptide is shown in green. Guanosine diphosphate (GDP) and aluminum fluoride are shown as stick representations, and Mg^2+^ ions are shown in dark green. Residues mutated in corresponding *V. alginolyticus* proteins are highlighted by blue (function reduced) or red (abolished) balls with the residue number of *Vibrio* protein. The putative catalytic site (R334 of *Vibrio* FlhF) is also indicated. For simplicity, the above residues are highlighted only in one protomer. In *Vibrio* FlhF, alanine substitution of the catalytic residue (R334A) did not affect its function, indicating that GTP binding, but not hydrolysis, is essential.

Sequence analysis revealed that FlhF is one of the three members of the signal recognition particle (SRP)-type guanosine triphosphate (GTP)ase subfamily of SIMIBI (signal recognition particle, MinD and BioD)-class nucleotide binding proteins [36]. The other two members of that family are the signal sequence binding protein Ffh and the SRP receptor FtsY, whose structures have already been solved (Figure 3a) [37]. As shown in Figure 3b, FlhF is composed of a basic N-terminal domain (B domain) followed by a conserved NG domain (regulatory N domain and GTPase G domain) [38]. Structural and biochemical analyses of FlhF have been carried out for *Bacillus subtilis* FlhF (hereafter, *Bs*FlhF). In 2007, the crystal structure of the *Bs*FlhF NG domain homodimer in complex with guanosine triphosphate (GTP) was solved [39], and later in 2011, the *Bs*FlhF homodimer in a complex with the peptide containing N-terminal 23 residues of *Bs*FlhG was reported (Figure 3c) [40]. That structure shares homology with Ffh and FtsY within the NG domain. Ffh and FtsY form a GTP-dependent heterodimer via their NG domains (Figure 3a), and its GTPase activity is coupled to their function in targeting a ribosome-nascent chain complex to the Sec machinery on the cytoplasmic membrane [37]. 

Because of the structural similarity, it would be plausible that the FlhF homodimer functions in the numerous/spatial regulation of flagella similar to the way the Ffh/FtsY heterodimer does. To test that idea, mutational analyses of *Vibrio alginolyticus* FlhF (hereafter *Va*FlhF) have been performed. Site-specific mutations were introduced into conserved GTPase motifs (I, III, and IV; Figure 3b,c). The results showed that two of those mutations abolish the FlhF polar localization, flagellation, and thereby motility (T306A and D439A) [41]. Other mutants showed a correlation between the levels of polar localization and the ability to produce flagella. Later on, a random mutagenesis of full-length *flhF* was performed but mutations that abolished polar localization, flagellation, and motility were isolated only on the GTPase motif IV (Figure 3c, T436M and E440K) [42]. These results indicate that the GTPase motif of FlhF is functionally important, and to facilitate polar flagellation, the polar localization of FlhF is required.

The GTPase activity of FlhF was first reported for the *Bacillus subtilis* protein [40]. The results showed that *Bs*FlhF alone had only a low basal GTPase activity, but it was stimulated by FlhG (firstly named YlxH in *B. subtilis*). Subsequent analysis revealed that an N-terminal region of FlhG, which is not conserved in the MinD/ParA ATPase family, was responsible for the FlhF stimulation [40]. The crystal structure of the FlhF/FlhG-peptide (23 amino acids) complex showed that the FlhG peptide formed a helix and bound near the catalytic site of FlhF (Figure 3c) [40]. Later on, stimulation of the GTPase activity of *Campylobacter* FlhF (*Cj*FlhF) and *Va*FlhF by their cognate FlhG proteins was reported [43,44]. Consistent with *Bs*FlhF, purified *Va*FlhF existed as a homodimer in the presence of GTP but as a monomer in the presence of GDP [44]. These results suggest that the GTP-bound *Va*FlhF homodimer functions as an active form at the cell pole to promote flagellation, similar to the GTP-bound FtsY/Ffh complex at the Sec machinery. If so, a defect in the catalytic site would result in the accumulation of GTP-bound FlhF at the cell pole and cause hyperflagellation. This idea was supported by evidence that such a mutation in *Cj*FlhF (R324A) abolished the GTPase activity and increased the hyperflagellated population of *Campylobacter* cells [43,45]. On the other hand, the corresponding mutation (R334A, Figure 3c) in *Va*FlhF abolished its GTPase activity but still allowed it to localize at the cell pole and led to the normal polar flagellation of *V. alginolyticus* cells [44]. Similar results were reported for *Vibrio cholerae* FlhF (*Vc*FlhF), showing that substitutions of putative catalytic residues had little effect on *Vc*FlhF function, which indicated that GTP binding, but not hydrolysis, is critical for *Vibrio* FlhF function [16]. The varied phenotypes of FlhF mutants among species may reflect diverse flagellation patterns (mono- or bi-polar flagellation) or flagellar function (e.g., rotational speed or motor power) [11,38].

How FlhF promotes polar flagellation remains unknown. One plausible idea is that FlhF acts on the initial step of assembly of the flagellar basal body. Flagellar assembly begins with the formation of basal rings (MS- and C-rings; Figure 1a) that house the flagellum-specific export apparatus, followed by the construction of axial structures (rod, hook, and associated outer ring structures), and then is completed with the filament and motor part assembly [46]. The MS-ring is believed to be one of the earliest structures assembled in a flagellum [47], and FlhF may facilitate flagellation by recruiting FliF, a membrane protein and MS-ring component, to the cell pole. This idea is supported by evidence that the polar localization of GFP-fused FliF is dependent on FlhF expression in *Vibrio cholerae* [16]. Mutational analysis revealed that the B and N domains are essential for recruitment of FliF to the cell pole. A nonfunctional *Vc*FlhF D367A mutant of the GTPase motif III was still able to recruit FliF to the cell pole, but it inhibited flagellar assembly, suggesting the involvement of *Vc*FlhF in the MS-ring formation. Further studies are required to address the role of FlhF in the promotion of flagellar assembly. 

## 4. FlhG Negatively Regulates Polar Flagellar Gene Expression

**Figure 4:**
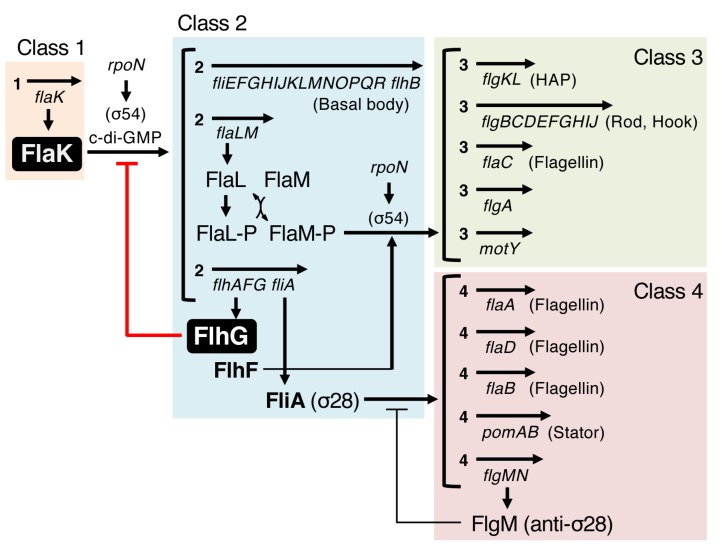
Proposed model for the regulation of *Vibrio alginolyticus* polar flagellar transcription hierarchy. This model is based on reports for *Vibrio cholerae* and *Vibrio parahaemolyticus* [54,55], whose flagellar genes are highly similar. The master regulator FlaK, which belongs to class 1 as a sole member, regulates downstream flagellar genes. FlaK activity is negatively regulated by FlhG, or FlhG may inhibit the transcription of *flaK*. The signaling molecule c-di-GMP also negatively regulates FlaK activity.

Assembly of a flagellum requires the synthesis of enormous amounts of protein components, including the long flagellar filament, and thus this process consumes large quantities of energy. Therefore, bacteria developed an efficient construction strategy—assembly occurs in a stepwise fashion to build from inner to outer structures, and is tightly coupled with flagellar gene transcription to provide necessary components at each step [46]. To achieve assembly-coupled transcription, flagellar genes are organized into a transcriptional hierarchy that is comprised of three to four classes of genes, with classification varying on species (Figure 4) [48,49]. On top of this hierarchy, a master regulator, which is usually the sole member of class 1, controls the expression of downstream flagellar genes. In *Pseudomonas aeruginosa*, *Vibrio cholerae* and *Vibrio parahaemolyticus*, the regulators FleQ [50], FlrA [51], and FlaK [52] have been identified as the master regulators, respectively. As one can imagine, hyperflagellation due to the flhG mutation demands a large amount of flagellar components, and indeed, a lack of FlhG caused the upregulation of flagellar gene expression [33,34]. In *Pseudomonas aeruginosa*, FlhG (named FleN in *Pseudomonas*) does not affect the expression of *fleQ* [33] but rather physically interacts with the FleQ protein to inhibit its transcriptional activity [53], whereas in *Vibrio cholerae*, FlhG represses the expression of *flrA* to downregulate flagellar gene expression [54]. Therefore, FlhG regulates flagellar biogenesis at the transcriptional level by negatively acting on the expression (for *flrA*) or activity (for FleQ) of the master regulator. However, our research group also found that the negative regulation of FlhG for flagellar biogenesis occurs at the post-translational level. Such a mechanism is reviewed in the next two sections.

## 5. HubP, the Third Regulator of Polar Flagellar Biogenesis

**Figure 5:**
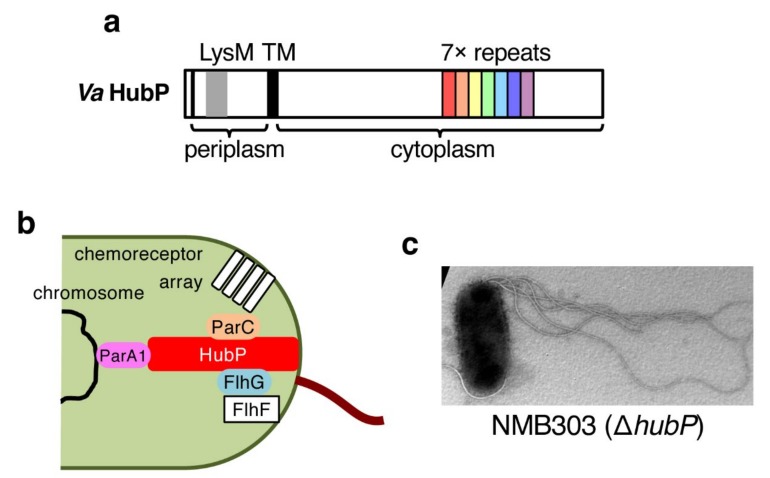
HubP, the third factor that regulates the number of polar flagella in *Vibrio alginolyticus*. (**a**) Domain structure of *V. alginolyticus* HubP, a single transmembrane protein of 1444 amino acids (≈159 kDa) with a large cytoplasmic region. The LysM domain in the N-terminal periplasmic region functions in anchoring HubP to the peptidoglycan layer. The cytoplasmic repeat sequence and its repetition numbers varies among *Vibrio* species. (**b**) HubP functions as the polar “hub”. HubP localizes at the cell pole and anchors three ParA-like proteins at its large cytoplasmic platform. FlhF has an intrinsic property to localize at the cell pole, but FlhG polar localization is dependent on HubP. (**c**) Electron micrograph of a NMB303 cell, the *hubP* deletion strain of *V. alginolyticus*. It generates multiple sheathed flagella at the cell pole.

HubP was first identified in *Vibrio cholerae* as a polar landmark protein that anchors three ParA-family proteins including FlhG [56]. HubP is conserved in *Vibrio* species [57], in *Shewanella* [30], and in some other gamma-proteobacteria, and shows similarity to FimV, a positive regulator for type IV pilus formation [58]. HubP is quite a large protein (1444 amino acids, ≈159 kDa for *V. alginolyticus* protein [57]), and has a single transmembrane segment with an N-terminal region placed in the periplasm (Figure 5a). The periplasmic LysM domain, which has been implicated in peptidoglycan binding, is important for the polar localization of HubP [56]. The large cytoplasmic C-terminal region contains 7–10 copies of the repeat sequence that interacts with ParA1, and FlhG is found to interact with the extreme C-terminus of HubP (Figure 5a,b) [56]. 

Although the deletion of *hubP* did not affect the polar flagellation of *V. cholerae* [56], the deletion of *hubP* in *V. alginolyticus* increased the number of polar flagella (Figure 5c) [57]. The level of hyperflagellation is stronger for the *flhG* mutant, which had more flagella per cell than the Δ*hubP* strain, and the additional deletion of *hubP* from the *flhG* mutant did not further increase the number of flagella [57]. These results indicate that HubP is also involved in regulating the number of polar flagella in *V. alginolyticus* to a certain level. How then is HubP involved in the biosynthesis of polar flagella? The *hubP* gene is not included in the polar flagellar gene cluster, and the endogenous chromosomal expression level of FlhG in the Δ*hubP* mutant is comparable to that in the wild-type strain [57], indicating that the expression of polar flagellar genes is not affected by the deletion of *hubP*. On the other hand, the polar localization of FlhG, but not FlhF, was abolished in the Δ*hubP* mutant as observed in *V. cholerae* [56,57]. These results suggest that FlhG localized at the cell pole negatively regulates flagellar biogenesis. If so, FlhG functions not only at the transcriptional level, but also at the post-translational level by localizing at the cell pole. In the next section, we describe the post-translational regulation of polar flagellar biogenesis by FlhG.

## 6. FlhG Is a MinD/ParA-Type ATPase

**Figure 6:**
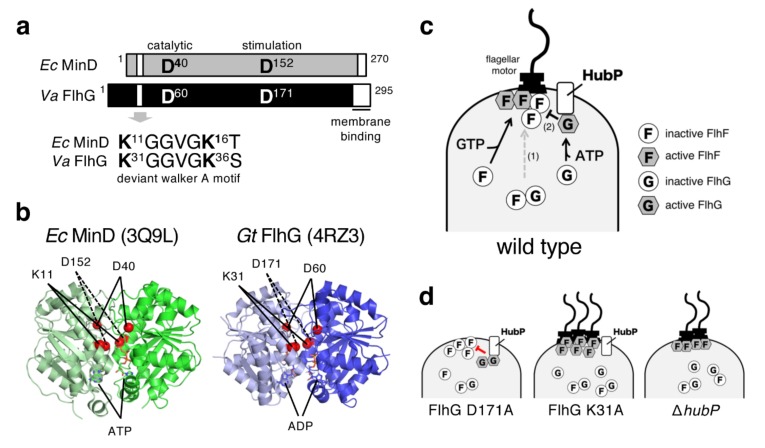
FlhG is a MinD/ParA-like ATPase that negatively regulates polar flagellar number. (**a**) Domain structures of *Escherichia coli* MinD (*Ec* MinD) and *V. alginolyticus* FlhG (*Va* FlhG). FlhG has a slightly longer N-terminal region than MinD, which functions in the stimulation of FlhF GTPase activity. (**b**) Crystal structures of *E. coli* MinD dimer in complex with ATP (PDB ID: 3Q9L [62]) and *Geobacillus thermodenitrificans* FlhG dimer in complex with adenosine diphosphate (ADP) (PDB ID: 4RZ3 [60]). ATP and ADP are shown by stick representations, and important conserved residues are colored red. (**c**) Model for the regulation of the number of polar flagella in *Vibrio alginolyticus*. GDP-bound FlhF and ADP-bound FlhG are in an inactive state, interact with each other and remain in the cytoplasm. When GTP is bound, FlhF becomes active and localizes at the cell pole to facilitate flagellation. Likewise, the ATP-bound active form of FlhG localizes to the cell pole via the landmark membrane protein HubP to inhibit FlhF activity. The inhibition of FlhF polar localization (1) and its activity (2) by FlhG optimizes the number of flagella into becoming a single one. (**d**) Working models to explain nonflagellated or hyperflagellated phenotypes of FlhG or HubP mutants of *V. alginolyticus*. FlhG D171A, a putative activated mutant, inhibits polar flagellation by more localization of FlhG at the cell pole. FlhG K31A, a nonfunctional mutant, causes hyperflagellation because it cannot localize at the cell pole. K31A also cannot inhibit FlaK so that more flagellar proteins are synthesized. The Δ*hubP* strain produces the wild-type level of flagellar proteins, but its polar flagellar number increases because FlhG cannot localize at the cell pole.

Sequence analysis revealed that FlhG (FleN) is classified as a MinD/ParA-type ATPase [38]. MinD is the ATPase component of the Min system that is involved in the spatial regulation of cell division [59]. It forms a homodimer in the presence of ATP and that homodimer binds to the membrane at the cell pole via the C-terminal amphipathic helix. MinD ATPase is then stimulated by MinE, and this hydrolysis induces the release of MinD from the membrane as a monomer. This ATP-dependent dimerization and polar localization is essential for the function of MinD. Meanwhile, FlhG is composed of a MinD/ParA-homologous domain and an N-terminal extension that stimulates FlhF GTPase [40]. All functionally important residues in MinD are conserved in FlhG, and both have a membrane binding sequence at their C-termini (Figure 6a). The crystal structures of *Geobacillus thermodenitrificans* FlhG [60] and *Pseudomonas aeruginosa* FleN (FlhG) [61] revealed that it is indeed a structurally close homolog of MinD (Figure 6b) [62]. Biochemical characterization of FlhG in *Geobacillus* has revealed that it has quite similar properties to MinD—an ATP-bound FlhG homodimer associates with the plasma membrane through its C-terminal amphipathic helix, and hydrolysis of ATP causes dissociation of FlhG from the membrane as a monomer [60]. Interestingly, deletion of *flhG* in *Campylobacter jejuni* caused more cell division at the polar region to form minicells [12]. Because *Campylobacter* species lack a Min system, FlhG may take over Min function to inhibit division at the cell pole. 

For *V. alginolyticus* FlhG, the purified protein alone exhibits a low basal ATPase activity, but it can be activated sevenfold by the D171A mutation [63]. The corresponding mutation, D152A of *E. coli* MinD, confers MinD insensitivity to MinE stimulation for ATPase activity [64]. As discussed in the previous section, FlhG at the cell pole inhibits flagellation, presumably by acting on the polar FlhF (Figure 6c). This active *Vibrio* FlhG D171A mutant localizes at the cell pole more strongly than wild-type FlhG and severely inhibits flagellation (Figure 6d) [63]. On the other hand, mutations at putative ATP binding residues in the deviant Walker A motif impair various properties of FlhG, such as its ATPase activity, polar localization, and negative regulator activity for flagellar biosynthesis, and thus confers the hyperflagellated phenotype (Figure 6d) [63]. 

Unexpectedly, a mutation at the catalytic residue (D60A) that abolishes ATPase activity but still allows ATP binding, only slightly affected FlhG function [63]. These results suggest that the ATP-dependent polar localization of FlhG is crucial for its negative regulator activity. Because the polar localization of FlhG is dependent on the landmark membrane protein HubP, we speculate that ATP-bound FlhG localizes at the cell pole via HubP and becomes active to directly inhibit FlhF at the cell pole (Figure 6c), and that the adenosine diphosphate (ADP)-bound inactive form interacts with cytoplasmic FlhF to interfere with its polar localization (Figure 6c) [57]. It should be noted that the number of polar flagella seem to not be determined primarily by the absolute amount of polar FlhF, as proposed in our first model (Figure 2b). The amount of FlhF at the cell pole was not increased by the deletion of hubP (hyperflagellation) and was not reduced by the overproduction of *flhG* (nonflagellation). Currently, we hypothesize that cytoplasmic FlhG works as a quantitative regulator that controls the amount of FlhF at the cell pole, and HubP-anchored polar FlhG works as a qualitative regulator that directly inhibits FlhF activity at the cell pole (Figure 6c,d) [57]. It should be noted that the FlhG mutant at the putative ATP binding site upregulated polar flagellar gene expression (thereby conferring the hyperflagellated phenotype) [63], which suggests that ATP binding is important for FlhG function to negatively regulate the master regulator FlaK. 

As described above, MinD/ParA-family ATPases are known to form dimers in complex with ATP (Figure 6b), and that dimerization allows them to bind to the cell membrane where they exhibit their activities [65]. Recently, whether *Vibrio alginolyticus* FlhG undergoes ATP-dependent dimerization was examined [66]. The results showed that purified FlhG or FlhG in *Vibrio* cell lysates appeared to exist as a monomer in the presence of ATP or ADP, which suggests that ATP does not induce its dimerization. These results raise the possibility that monomeric FlhG can function *in vivo*, or alternatively, that an ATP-dependent FlhG dimer is unstable compared to other family member proteins and requires other factor(s) to stabilize the dimer structure [66]. In addition, mutations at the putative ATP binding or catalytic sites did not affect the elution profile of FlhG in size exclusion chromatography (eluted as a monomer regardless of the nucleotides), but the ATPase-active FlhG mutant (D171A) eluted slightly earlier in the presence of ATP but not ADP, presumably due to a subtle conformational change. Because the purified D171A mutant tends to aggregate in the presence of ATP, we speculate that ATP-bound active FlhG has a fragile conformation that causes its aggregation, but interactions with other proteins at the cell pole (most likely, HubP) prevent that aggregation and exhibit its function to inhibit FlhF activity [66]. 

It should be noted that *Geobacillus* and *Shewanella* FlhG have been shown to bind to the flagellar C-ring proteins FliM and FliY (FliN ortholog) in a nucleotide-independent manner [60]. Moreover, in the presence of ATP and lipids, *Geobacillus* FlhG (presumably in the dimer form) can activate FliM/FliY to assemble with another C-ring protein FliG in vitro [60]. These results raise the possibility that FlhG delivers C-ring proteins to the nascent flagellum, but this is puzzling because in this case FlhG functions as a positive regulator that promotes flagellar assembly. An alternative possibility is that FlhG binding to these proteins blocks the assembly of a nascent flagellum. Further analyses are required to clarify the enigmatic function of FlhG in the biogenesis of flagella.

## 7. SflA Represses Flagellar Biogenesis in the Absence of FlhF and FlhG

**Figure 7:**
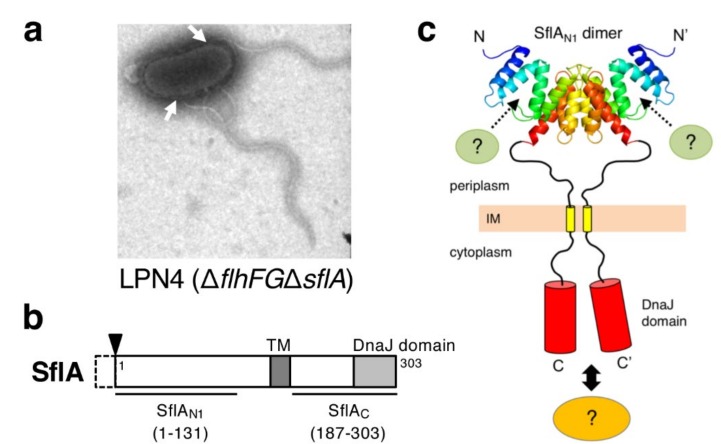
SflA represses lateral flagellation in the absence of both FlhF and FlhG in *Vibrio alginolyticus*. (**a**) Electron micrograph of a LPN4 cell, the Δ*flhFG*Δ*sflA* mutant. The sheathed flagella were formed at lateral positions, as indicated by the white arrows. (**b**) Domain structure of SflA. SflA is synthesized as a precursor with an N-terminal signal sequence that is cleaved during maturation (the cleavage site is shown as a black arrowhead). (**c**) Model of the molecular architecture of the SflA dimer. Unknown binding partner proteins bind to the concave surface of the N-terminal tetratricopeptide repeat (TPR)/Sel1-like repeat (SLR) domain of SflA, as indicated by the broken arrows. The DnaJ domain is activated by the binding signal transmitted through the membrane, and interacts with an unknown partner protein to suppress the formation of the sheathed flagellum at peritrichous cell surfaces or promote it at the cell pole. IM, inner membrane.

In *V. alginolyticus*, the deletion of both flhF and flhG from the strain VIO5 (wild-type for polar flagellum) resulted in a nonflagellated phenotype, but a very small fraction of the population produced several sheathed flagella at lateral positions [34]. The motile pseudo-revertants were isolated from the strain deleted for both *flhF* and *flhG* (Δ*flhFG*), which forms peritrichous flagella in the majority of cells [67]. Because these flagella were covered with a sheath and contained flagellins of the polar flagellum, the suppressor mutations increased the population of cells that produces multiple polar flagella at lateral positions. The mutation was mapped to a previously uncharacterized gene named *sflA* (suppressor of Δ*flhFG*) and the deletion of *sflA* from the Δ*flhFG* strain showed the suppression phenotype (Figure 7a) [68]. The *sflA* is specific for *Vibrio* species and is predicted to encode a single transmembrane protein (the mature protein contains 303 amino acids, ≈35 kDa) with its N-terminal region located at the periplasm. The cytoplasmic C-terminal region contains a DnaJ domain conserved in chaperone family proteins (Figure 7b) [69]. As with *hubP*, *sflA* is not included in known polar flagellar gene clusters, and therefore seems not to function specifically in flagellar biogenesis. The SflA protein was detected in the wild-type strain, but deletion of *sflA* from the wild-type strain did not affect polar flagellation and motility [68]. Overexpression of the C-terminal soluble region containing the DnaJ domain (SflA_C_, Figure 7b) suppressed the lateral flagellation of the Δ*flhFG*Δ*sflA* strain [70]. SflA fused with fluorescent protein showed a HubP-dependent polar localization in the presence of FlhF and FlhG, but was observed at polar and lateral positions in Δ*flhFG* cells [70]. These observations suggest that SflA localizes with flagella and that SflA_C_ represses the flagellar initiation in Δ*flhFG* cells by a currently unknown mechanism [70]. FlhF seems to be dominant over SflA in flagellation at the cell pole and voids the function of SflA. Recently, the crystal structure of SflA was solved for the N-terminal 131 residues (SflA_N1_; Figure 7b,c) [71]. The core of SflA_N1_ forms a domain-swapped dimer with a tetratricopeptide repeat (TPR)/Sel1-like repeat (SLR) motif, which is often found in domains responsible for protein–protein interactions in various proteins. SflA_N1_ has a characteristic positively charged area at the surface, and alanine substitutions in that area reduced the SflA function of inhibiting flagellation in Δ*flhFG* cells, which suggests that SflA_N1_ binds to an unknown partner protein and that the binding signal is transmitted to SflA_C_ to suppress the formation of the sheathed flagellum at lateral positions (Figure 7c) [71]. 

## 8. Conclusions and Perspectives

**Figure 8:**
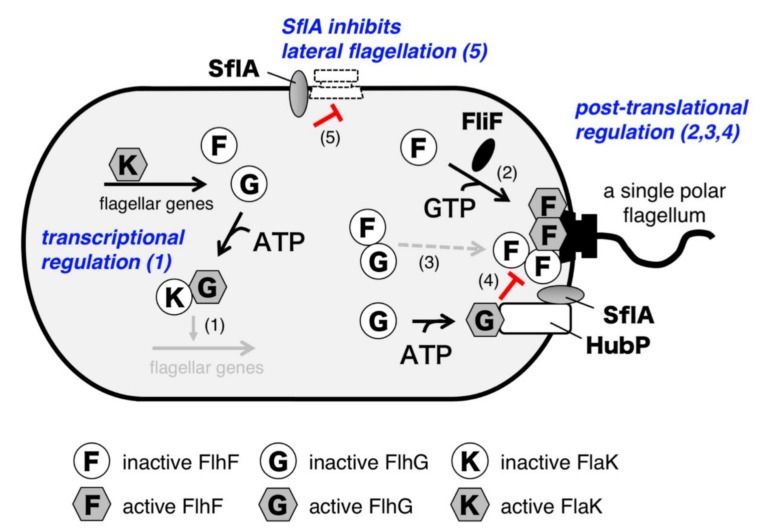
Model for the regulation of the biogenesis of a single polar flagellum in *Vibrio alginolyticus.* Five regulatory mechanisms are involved in this proposed model. When flagellar components are synthesized and accumulate, FlhG negatively acts on the master regulator FlaK to downregulate the expression of polar flagellar genes (1). FlhF forms a homodimer in complex with GTP and localizes at the cell pole. FlhF facilitates the accumulation of the MS-ring protein FliF at the cell pole and thereby promotes the MS-ring formation (2). Inactive FlhF and FlhG interact with each other and remain in the cytoplasm (3), and GTP-bound FlhG is activated by HubP at the cell pole and negatively acts on FlhF to inactivate the FlhF dimer at the cell pole (4). In addition to the transcriptional regulation, these post-translational mechanisms optimize polar FlhF activity that allows the cell to generate only a single polar flagellum. Meanwhile, SflA inhibits sheathed flagellar formation at lateral positions by negatively acting on its assembly step (5). FlhF activity is dominant over SflA at the cell pole, so that the effect of SflA, localized at the cell pole via HubP, is suppressed.

To summarize all the insights presented in this review, we would like to propose a model for the biogenesis of a single polar flagellum in *Vibrio alginolyticus* (Figure 8). At least five factors are involved in this precise control: FlhF, FlhG, HubP, FlaK, and SflA. When cells are growing, the polar flagellar genes are transcribed in a cascade fashion. The class 1 master regulator FlaK activates expression of the class 2 genes, including *flhF*, *flhG*, and those for flagellar basal body components such as the MS-ring protein FliF. After it accumulates, FlhG negatively acts on the master regulator FlaK to shut off the expression of polar flagellar genes. This temporal regulatory mechanism prevents the unnecessary use of energy required for hyperflagellation. FlhF then forms a homodimer in complex with GTP and localizes at the cell pole. The polar localization of FlhF facilitates the accumulation of the MS-ring protein FliF at the cell pole, and thereby promotes the MS-ring formation there. Inactive ADP-bound FlhG binds to GDP-bound inactive FlhF and interferes with its polar localization in the cytoplasm, whereas ATP-bound FlhG is able to associate with HubP at the cell pole. HubP induces the structural change of FlhG to become an active form, in which the active site for ATP hydrolysis and binding interface for FlhF are constituted. This allows FlhG to directly inhibit the FlhF dimer, presumably by stimulating GTP hydrolysis. These four regulatory steps (indicated as “1” to “4” in Figure 8) together optimize FlhF activity at the cell pole to generate only a single polar flagellum. Meanwhile, initiation of sheathed flagellar assembly could occur at lateral positions once the MS-ring and flagellar specific export apparatus are assembled. However, in such a case, SflA inhibits the completion of flagellar assembly (indicated as “5” in Figure 8). Therefore, SflA also participates in regulation of flagellar biogenesis at the cell pole.

Many questions remain to be solved. For example, how does FlhF promote MS-ring assembly? The structural similarity suggests that GTP-bound FlhF homodimer may function similar to the GTP-dependent heterodimer of the signal recognition particle (SRP) and its receptor (SR). However, the Δ*flhFG*Δ*sflA* strain can form sheathed flagella at lateral positions [68], and thus FlhF is not essential to insert nascent FliF, the MS-ring protein, in membranes. Therefore, FlhF seems to function in delivering or specifically inserting FliF to the cell pole. Alternatively, FlhF may facilitate the MS-ring assembly step at the cell pole. Further, how FlhG works at the cell pole is still largely unknown. It can function as a monomer or, alternatively, ATP-bound FlhG dimer may be stabilized at the cell pole to inhibit FlhF. Because the majority of *Bacillus* FlhG *in vivo* is reported as being highly mobile [60] but *Vibrio* FlhF is observed at the base of an assembled polar flagellum [34], FlhG may dissociate from the cell pole after the ATP hydrolysis, although FlhF somehow remains at the flagellated cell pole. To clarify the functions of FlhF and FlhG in flagellar biogenesis, their structural and biochemical properties must be understood on the basis of their temporal behavior at the cell pole. It should also be remembered that it remains enigmatic as to how SflA and FlaK recognize the flagellation state of cells. Altogether, it is fascinating that even simple organisms such as bacteria have such complex mechanisms to precisely control the temporal and subcellular positioning of biomolecules. The journey to elucidate the mechanism of how a single polar flagellum is generated by bacteria is a challenging one that will be exciting to explore.
